# Bacterial Blight Induced Shifts in Endophytic Microbiome of Rice Leaves and the Enrichment of Specific Bacterial Strains With Pathogen Antagonism

**DOI:** 10.3389/fpls.2020.00963

**Published:** 2020-07-23

**Authors:** Fenghuan Yang, Jie Zhang, Huaying Zhang, Guanghai Ji, Liexian Zeng, Yan Li, Chao Yu, W. G. Dilantha Fernando, Wen Chen

**Affiliations:** ^1^State Key Laboratory for Biology of Plant Diseases and Insect Pests, Institute of Plant Protection, Chinese Academy of Agricultural Sciences, Beijing, China; ^2^Ottawa Research & Development Centre, Science & Technology Branch, Agriculture and Agri-Food Canada, Ottawa, ON, Canada; ^3^College of Plant Protection, Yunnan Agricultural University, Kunming, China; ^4^Plant Protection Research Institute, Guangdong Academy of Agricultural Sciences, Guangzhou, China; ^5^Department of Plant Pathology, College of Plant Protection, China Agricultural University, Beijing, China; ^6^Department of Plant Science, University of Manitoba, Winnipeg, MB, Canada

**Keywords:** *Oryza sativa* L., phytomicrobiome, plant endophytes, *Pantoea* spp, bacterial leaf blight of rice

## Abstract

The endophytic microbiome plays an important role in plant health and pathogenesis. However, little is known about its relationship with bacterial blight (BB) of rice caused by *Xanthomonas oryzae* pv. *oryzae* (*Xoo*). The current study compared the community compositional structure of the endophytic microbiota in healthy and BB symptomatic leaves of rice through a metabarcoding approach, which revealed BB induced a decrease in the alpha-diversity of the fungal communities and an increase in the bacterial communities. BB-diseased rice leaves were enriched with saprophytic fungi that are capable of decomposing plant cell walls (*e.g. Khuskia* spp. and *Leptosphaerulina* spp.), while healthy rice leaves were found to be significantly more abundant with plant pathogens or mycotoxin-producing fungi (*e.g. Fusarium*, *Magnaporthe*, and *Aspergillus*). The endophytic bacterial communities of BB-diseased leaves were significantly enriched with *Pantoea*, *Pseudomonas*, and *Curtobacterium*, strains. *Pantoea* sp. isolates from BB leaves are identified as promising candidates for the biocontrol of BB for their ability to inhibit *in vitro* growth of *Xoo*, suppress the development of rice BB disease, and possess multiple PGP characteristics. Our study revealed BB-induced complexed changes in the endophytic fungal and bacterial communities of rice leaves and demonstrated that BB-associated enrichment of some endophytic bacterial taxa, *e.g. Pantoea* sp. isolates, may play important roles in suppressing the development of BB disease in rice.

## Introduction

Rice (*Oryza sativa* L.) is an important cereal crop consumed as a staple food by half of the world’s population (Fairhurst and Dobermann, 2002). Rice disease epidemics not only directly result in yield reduction and thus threaten local and global food security, but also substantially increase the cost of disease management and overall rice production. Bacterial leaf blight of rice (BB) is a disease caused by the Gram-negative bacterium *Xanthomonas oryzae* pv. *oryzae* (*Xoo*), which typically invades the rice plant through wounds or hydathode water pores, moves and colonizes the xylem vessels of leaves, and results in tannish-gray to white lesions along the veins (Nino-Liu et al., 2006). BB may lead to 20–50% yield loss and up to 100% during an epidemic. Bacterial blight has become one of the most severe and prevalent rice diseases globally (Leach et al., 1992; Adhikari et al., 1995). Strategies for managing this disease include cultivating disease-resistant rice varieties, application of chemical pesticides, and the use of antagonistic bacteria, many of which were recovered from the rhizosphere, phyloplane, or endophytic tissues of rice or other plant sources (Gnanamanickam, 2009; Gangwar, 2013; Krishnan et al., 2014; Chung et al., 2015).

Plant endophytic microorganisms, *e.g.* bacteria, archaea and fungi, can colonize their hosts internally, with some being inherited through generations (vertically transmitted), while most are acquired from the environment (horizontally transmitted) (Leach et al., 2017). Therefore, the endophytic microbiota are plant genotype-specific and reflect the adaptation of the host to habitat conditions relevant to, for example, the presence of phytopathogens or accessibility of nutrients (Frank et al., 2017). However, distinct endophytic microbiota of different plant species, often harbor microorganisms with similar functional traits yet phylogenetically distant. For instance, the nitrogen-fixing bacteria revealed from rice plants were *Azoarcus* spp. (in Betaproteobacteria), while those from maize were *Azospirillum* spp. (in Alphaproteobacteria) (Kandel et al., 2017). Previous studies have indicated that the diversity and compositional structure of rice endophytic microbiome vary with plant genotype, tissue type, growth stage, and environmental conditions (Wang et al., 2016a; Walitang et al., 2018; Qin et al., 2019). *In vitro* experiments have shown that endophytic isolates (*e.g.* bacterial strains of *Bacillus*, *Klebsiella*, and *Streptomyces*, and fungal strains of *Chaetomium globosum*, *Penicillium chrysogenum*, and *Azospirillum* sp. B510) could effectively suppress the growth of some phytopathogenic bacteria and/or fungi (such as *Xoo*, *Burkholderia glumae*, *F*. *oxysporum*, *Rhizoctonia solani*, *Magnaporthe grisea*) (Naik et al., 2009; Ikeda et al., 2010; Ji et al., 2014; Chung et al., 2015). In addition, endophytes with plant growth promoting (PGP) attributes may induce broad spectrum resistance to phytopathogens by stimulating induced systemic resistance, competing for nutrients, and producing phytohormones or antagonistic allelochemicals (Compant et al., 2005). Rice endophytes, such as the strains of *Burkholderia* sp., *Enterobacter* sp., *Pantoea* sp., *Pseudomonas* sp., and *Sphingomonas* sp., have exhibited the ability to produce phytohormones such as indole-3-acetic acid (IAA) and siderophore (Wang et al., 2016b).

There is an increased attention for the interactions between pathogens and the residential microbiota as well as interactions of different plant pathogens. Previous studies have indicated that the invasion of phytopathogens induces changes in the composition and the associations of the internal microbial habitants and affects the behavior of other plant pathogens (Wang et al., 2017; Li et al., 2019). For example, the microbiome of the Huanglongbing (HLB)-diseased citrus was depleted of beneficial bacteria with PGP traits, while the infected host plants were more susceptible to infection by *Phytophthora*, *Colletotrichum acutatum*, and *X. citri* (Yang et al., 2016; Wang et al., 2017; Zhang et al., 2017). It was also found that potato common scab could modulate the composition and function of the geocaulosphere microbiome; severe infection resulted in low diversity, sparse co-occurrence network and high scab phytotoxin biosynthetic gene copies (Shi et al., 2019). These studies thus open the avenue for studying the roles of phytomicrobiome during plant disease development, for identifying synergistic interactions between pathogens and host endophytes, and for revealing naturally occurring biocontrol agents for plant diseases.

While the composition and some functions of the endophytic microbiota of rice have been studied in great detail, little is known about how it is affected by the occurrence of diseases, especially the economically important BB. The objective of this study was to compare the endophytic microbiota of healthy and BB leaves of rice, through which to identify microbial indicators that potentially facilitate or suppress the development of BB in rice. The endophytic microbiota in healthy and BB leaves of rice was comparatively profiled through metabarcoding the bacterial 16S rRNA gene and the fungal internal transcribed spacer (ITS) region. The BB induced enrichment of endophytic strains of *Pantoea*, which were isolated and assessed for their putative roles in BB development. Our study provides novel insights into the BB-induced changes in rice endophytic microbiome and the potential roles of enriched endophytes in suppressing the disease in BB occurrence.

## Materials and Methods

### Sampling of Rice Leaves

The purpose of this study was to investigate the generalized impact of BB on rice endophytic microbiota, and therefore, we selected nine different rice cultivars (n = 9) with different BB-resistance levels for this study. Among these cultivars, four *O. sativa* L. ssp. japonica cultivars and two hybrid cultivars (**Table 1**) were planted in March 2017 in an experimental farm (Field1, (N 24.4°, E 98.5°)) that was managed by the Plant Protection Station in Mangshi, Yunnan province, China. When the rice plants of each cultivar had reached the tillering stage (August 6, 2017), the leaves of half of the plants were artificially inoculated with *Xoo* by pricking with a needle, while the other half of the plants were un-inoculated. Three hybrid cultivars (**Table 1**) were planted and managed by the local farmers in Mangshi [Field2–4, (N 24.4°, E 98.5°)]. Rice plants in Fields 2, 3, and 4 were naturally infected with BB pathogen. The healthy leaves without the BB symptoms and BB-diseased rice leaves of each rice cultivar were collected on August 20, 2017, which were used for the extraction of endophytic microbiome genomic DNA and for the isolation of *Xoo* strains. For isolating the endophytic bacterial strains, eight BB leaves of rice were harvested from the farmer fields of the Yunnan (N25°35′, E 100°13′) and Guangdong (N 23°13′, E 117°19′) provinces, China, in October 2018.

**Table 1:** Rice cultivars used in this study.

Samples	Variety	Origin	Resistance to *BB*	Location
A	*O. sativa* ssp. *japonica*	*O. sativa* ssp. *japonica*	Sensitive to *BB*	Field1
B	‘IR24’	*O. sativa* ssp. *japonica*	Sensitive to *BB*	Field1
C	‘IRBB4’	*O. sativa* ssp. *japonica*	Resistant: Xa4 gene	Field1
D	‘IRBB5’	*O. sativa* ssp. *Japonica*	Resistant: Xa5 gene	Field1
E	‘DianShan 2’	Chinese hybrid rice	unknown	Field1
F	‘DianLong201’	Chinese hybrid rice	unknown	Field1
G	‘Tianyouhuazhan’	Chinese hybrid rice	unknown	Field2
H	‘Yiyou673’	Chinese hybrid rice	unknown	Field3
I	‘Jingliangyou’	Chinese hybrid rice	unknown	Field4

Location of Field1: MangShi, Yunnan Province, China; Location of Field2, 3 &4: Mangshi, Yunnan Province, China.

### Preparation of Rice Leaves

The surface of each leaf was sterilized with 70% ethanol for 1 min and shaken in 1.2% (w/v) NaClO solution for 15 min. The leaf was then washed three times with sterile distilled water with shaking (15 min each time). The DNA of the surface microorganisms was removed by ultrasonication for 10 min. The sample was then washed with sterile distilled water three times. Each leaf sample was stored individually at −80°C until further processing.

### DNA Extraction and Metabarcoding

Total genomic DNA from each leaf sample was extracted using the CTAB/SDS method (Lutz et al., 2011). DNA concentration and purity were assessed on 1% agarose gels. The DNA pellet was diluted to 1 ng/μl using sterile water. Sequencing library preparations and Illumina HiSeq sequencing were conducted at Novogene Inc. (Beijing, China). The V4–V5 regions of the bacterial 16S ribosomal RNA gene were amplified using the 515F (5′-GTGCCAGCMGCCGCGG-3′)/907R (5′-CCGTCAATTCMTTTRAGTTT-3′) primer set, while the fungal ITS1 region was amplified using the ITS1F (5′-GCATCGATGAAGAACGCAGC-3′)/ITS1R (5′-TCCTCCGCTTATTGATATGC-3′) primer set. The high-throughput sequencing was carried out on an Illumina HiSeq2500 platform and 250 bp paired-end reads were generated.

### Sequencing Data Processing

Demultiplexed raw paired-end sequences were trimmed by Trimmomatic (version 0.32) (Bolger et al., 2014) to remove the adapters, primer sequences, and low-quality reads. The remaining reads were merged using FLASH (version 1.2.7) with a minimum overlap of 30 bp and a 3% maximum difference in the overlap region (Magoč and Salzberg, 2011). Chimeric sequences were removed using UCHIME (Edgar et al., 2011) against the “Gold” database (version microbiomeutil-r20110519, drive5.com/uchime) for the 16S rRNA gene metabarcodes and against the UCHIME ITS1 database downloaded from https://unite.ut.ee/ (version 7.2, release date 2017-06-28) for ITS1 metabarcodes. All remaining metabarcodes were clustered into operational taxonomic units (OTUs) at 97% identity cut-off using UPARSE (version 7.0.1001) (Edgar, 2013). The sequence with the highest frequency in an OTU was selected as the representative sequence, which was assigned to a taxonomic lineage using RDP classifier (version 2.2) by comparing with the SILVA database (v13_5) (McDonald et al., 2012) for the 16S rRNA gene V4–V5 metabarcodes or with the UNITE fungal ITS database (version 8.0, release date 2018-11-18) (Abarenkov et al., 2010) for the fungal ITS1 metabarcodes. The singletons and unassigned OTUs were removed from the OTU tables. Multiple sequence alignments were performed using MUSCLE (version 3.8.31) to obtain the phylogenetic relationships of OTUs (Edgar, 2004).

### Statistical Analysis and Visualization

All statistical analyses were carried out in the R environment (version 3.4.3) (R Core Team, 2017). The OTU tables were rarefied to the same sample size. The OTUs or taxonomic abundance matrices were transformed to relative abundances when required. Alpha diversity indices were calculated using the “OTU diversity” function from the “RAM” package (Chen et al., 2017). The Shannon (SH) and Simpson (S) indices were converted to true diversities (SH-TD, S-TD) as suggested by Jost (2006). The FAPROTAX (Script version 1.1) (Louca et al., 2016) and FUNGuild v1.0 database (Nguyen et al., 2016) were used to predict the functional guilds of the bacterial or fungi communities, respectively. The alpha-diversity indices and community data were subjected to centered log-ratio transformation (CLR) (Gloor et al., 2017) prior to statistical analysis. The impacts of BB-resistance level of rice varieties and leaf health status (healthy *vs.* diseased) on the alpha-diversity of microbial communities were evaluated by multiple linear regression models (MLR), while their impacts on the community compositional structure were evaluated by ANOSIM and redundancy analysis (RDA) in vegan package (Preacher et al., 2006; Oksanen et al., 2018). Function *compare_means* (“anova” for global assessment and “t.test” for pairwise comparison) in R package ggpubr was used to perform multiple mean comparisons of the abundance of microbial taxa between treatment groups. Heatmaps were created using the *pheatmap* function in the pheatmap R package (Kolde, 2015).

### Isolation and Identification of Endophytic Bacteria

The *Xoo* strains were isolated from the BB-diseased leaves of rice plants (**Supplementary Figure S1**) grown in Mangshi, while the other bacterial endophytes were isolated from *Xoo*-infected leaves of rice collected from Yunnan and Guangdong provinces. All rice leaves were surface-sterilized with 70% ethanol for 1 min and shaken in 1.2% (w/v) NaClO solution for 15 min. The leaves were then washed three times with sterile distilled water with shaking (15 min each). The water collected from the final washing step was used as the negative control. The leaf tissues were then homogenized using a plastic drill. For isolating endophytic bacterial strains, the suspension of the leaf tissues and the ‘washed’ water (negative control) were spread onto LB plates, which were incubated at 37°C for up to one week. For the isolation of *Xoo*, the leaf tissue suspension was spread on peptone sucrose agar (PSA) medium plates (Yang et al., 2012) and incubated at 28°C for one week. The colonies on the plates were picked and re-streaked several times to obtain pure isolates. Bacterial strains were stored at −80°C for further analysis. The identification of isolated bacterial strains was conducted by amplifying the16S rRNA gene of the pure cultures using the primer pairs 27f/1492R (Morontabarrios et al., 2018) or the primer OSF1/OSR1 for *Xoo* (Tian et al., 2014). The PCR products were purified and then sequenced. The sequences were searched by BLASTn against the GenBank Nucleotide (nt) database for the identification of the isolates. Multiple-sequence alignments were carried out using the DNAMAN software (version 6.0; LynnonBioSoft, Canada). The neighbor-joining tree was reconstructed using MEGA7 (v3.1/3.0 aLRT) (Saitou and Nei, 1987; Kumar et al., 2016). The full-length 16S rRNA gene sequences have been deposited in the GenBank with the accession numbers listed in **Table 2**.

**Table 2:** The bacterial endophytes isolated from the BB-diseased leaves of rice.

Bacteria Isolates	Accession NCBI ^A^	Closest Type Strain ^C^	Reference Sequence ^D^	Reference Resource	Taxonomy (Phylum)	Identities (%)
**Zhefang Town, Mangshi, Yunnan Province, China**						
ZFZa	MK578268	*Pantoea ananatis* strain PP1	KM675660.1 1	fruit, pathogen	Proteobacteria	99.37%
ZFZb	MK578271	*Micrococcus* sp. strain E4	MG963203.1	tomato seed, endophyte	Actinobacteria	100%
ZFZc	MK578270	*Microbacterium testaceum* StLB037	AP012052.1	potato leaf, endophyte (Morohoshi et al., 2011)	Actinobacteria	99.90%
ZFZd	MK578269	*Pantoea ananatis* strain PA	MK578186.1	paddy, pathogen	Proteobacteria	99.50%
**Mangrui road side, Yunnan Province, China**						
MRDDa	MK578272	*Pantoea agglomerans* strain S33	AY741162	rice, endophyte	Proteobacteria	99.64%
MRDDb	MK578273	*Enterobacter mori* strain YHBG16	MG516182.1	*Tripterygium wilfordii* root, endophyte	Proteobacteria	99.80%
MRDDc	MK578274	*Curtobacterium luteum* strain VRI6-A1,	KY882069.1	groundnut leaf, endophyte (Krishnamoorthy et al., 2018)	Actinobacteria	99.90%
MRDDd	MK578275	*Micrococcus luteus* strain AU C5.2	KY775492.1	marine macroalgae (Leiva et al., 2015)	Actinobacteria	100%
MRDDe	MK578276	*Pseudomonas* sp. JXR26	KP980602.1	wild rice, endophyte	Proteobacteria	99.90%
MRDDf	MK578277	*Microbacterium* sp. strain HBUM179310	KR906278.1	*Gynura* medicinal plant, endophyte	Actinobacteria	100%
**Manghe Village, Mangshi, Yunnan Province, China**						
MSMHa	MK578278	*Pantoea agglomerans* strain CFSAN047153	CP034469.1	‘Rome’ apple cultivar leaf, endophyte	Proteobacteria	99.40%
MSMHb	MK578279	*Micrococcus* sp. strain HBUM179241	KR906506.1	*Gynura* medicinal plant, endophyte	Actinobacteria	100%
MSMHc	MK578280	*Microbacterium* sp. Fse46	KJ733898.1	wild rice seed, endophyte	Actinobacteria	100%
MSMHe	MK578281	*Pantoea agglomerans* strain CFSAN047153	CP034469.1	‘Rome’ apple cultivar leaf, endophyte	Proteobacteria	99.20%
MSMHg	MK578282	*Pseudomonas* sp strain JXR2	KP980602.1	rice, endophyte	Proteobacteria	100%
**Nanjian Village, Zhefang Town, Mangshi, Yunnan Province, China**						
MSZFNJa	MK578283	*Pantoea vagans* strain Os_Ep_PSA_12	MN932345	rice panicle, endophyte	Proteobacteria	99.93%
MSZFNJc	MK578284	*Micrococcus luteus* strain AU C5.2	KY775492.1	macroalgae, endophyte	Actinobacteria	100%
MSZFNJd	MK578285	*Microbacterium testaceum* StLB037,	AP012052.1	potato leaf, endophyte (Morohoshi et al., 2011)	Actinobacteria	99.90%
**Guangnong Village, Zhefang Town, Mangshi, Yunnan Province, China**						
MSZFGNa	MK578289	*Paenibacillus polymyxa* strain CR9	KR780413.1	wild rice, endophyte	Firmicutes	99.80%
MSZFGNb	MK578297	*Pseudomonas* sp strain JXR2	KP980602.1	rice, endophyte	Proteobacteria	99.90%
MSZFGNc	MK578286	*Pantoea ananatis* strain EM2-53	MT212809	Rice leaf, endophyte	Proteobacteria	99.42%
MSZFGNd	MK578287	*Curtobacterium citreum* strain BAB-7159	MF319766.1	cauliflower leaf, endophyte	Actinobacteria	99.90%
MSZFGNf	MK578288	*Curtobacterium albidum* strain RTO	MK014287.1	rice, endophyte	Actinobacteria	100%
Baiyun District, Guangzhou, Guangdong Province, China						
GDGZBYa	MK578290	*Pantoea ananatis* strain Os_Ep_VSA_42	MN932327	rice panicle, endophyte	Proteobacteria	99.93%
GDGZBYb	MK578291	*Microbacterium* sp strain YNA115	JQ071519.1	rice root, endophyte	Actinobacteria	99.90%
GDGZBYc	MK578292	*Agrobacterium larrymoorei* strain UCCB	MH198279.1	*Ocimum Sanctum* leaf, endophyte	Proteobacteria	99.90%
**Yangchun, Guangdong Province, China**						
GDYCa	MK578293	*Pantoea ananatis* strain OsEnb_PLM_L19.1	MN889261.1	rice leaf, endophyte	Proteobacteria	99.93%
GDYCb	MK578294	*Curtobacterium luteum* strain JXS1	KP980576.1	rice, endophyte	Actinobacteria	100%
GDYCc	MK578295	*Microbacterium hydrothermale* strain BPSAC84	MK696251.1	medicinal plant, endophyte (Passari et al., 2019)	Actinobacteria	100%
GDYCd	MK578296	*Paenibacillus hunanensis* strain KT3	LC026005.1	rice seed, endophyte	Firmicutes	99.80%

### Inhibition of *Xoo* by Endophytic Bacterial Isolates

The antimicrobial activities of the endophytic bacteria were evaluated through zone of inhibition tests. The *Xoo* strain PXO99^A^ (Hopkins et al., 1992) cells grown in liquid M210 media (Yang et al., 2012) were re-suspended in sterile distilled water at OD_600_ = 0.8 and then inoculated on solid PSA plates by sterilized spreaders. The endophytic bacterial isolates were grown for 72 h at 37°C on LB plates. The agar blocks with endophytic bacteria were placed inversely on the PSA plate with *Xoo*. The *Xoo*-plate inoculated with blank agar blocks was used as the negative control. The plates were incubated for 48–72 h at 28°C. The inhibition zones of endophytic bacteria were measured and recorded. Each screening test was repeated three times.

### *In Vitro* Assessment of Plant Growth-Promotion Attributes

The plant growth-promoting traits of bacterial endophytic strains were tested by carrying out the following bioassays as described previously: the production of 1) indole acetic acid (IAA) (Bric et al., 1991), 2) hydrogen cyanide (HCN) (Mehnaz et al., 2010), and 3) exopolysaccharide (EPS); 4) solubilization of inorganic phosphate (Andreolli et al., 2016); 5) 1-aminocyclopropane-1-carboxylic acid (ACC) deaminase activity (Lacava et al., 2004; Glick, 2014); and 6) cell motility (Yang et al., 2012). All experiments were repeated at least three times.

### *In Planta* Evaluation of *Pantoea* sp. Isolates on the Development of BB Disease

Rice cultivar *Oryza sativa* L. ssp. indica ‘IR24’ was used for pathogenicity assays. First, *Xoo* PXO99^A^ was cultured in M210 media, while endophytic bacterial strains (*Pantoea* sp. ZFZa, GDYCa, MSMHa, and *Curtobacterium* sp. GDYCb) isolated from the current study were cultured in LB at 28°C until OD_600_ = 0.8. The cells of each bacterial strain were re-suspended in sterile distilled water. Each endophytic bacterial strain was mixed with *Xoo* PXO99^A^ at a concentration of 1:10, respectively. Each mixed inoculant was inoculated on to ≥10 rice leaves using the leaf clipping method (Ray et al., 2000). The lesion length of the leaves was scored on the fourteenth day post-inoculation (dpi). At least ten rice leaves on three plants were used for each endophytic bacterial strain, and all experiments were repeated three times.

### Sequencing Data Accessibility

The raw Illumina HiSeq paired-end sequences have been deposited at the NCBI’s sequence read archive (SRA) in BioProject ID PRJNA534010 with accession numbers SRR8948950–SRR8949003 and PRJNA533998 with accession numbers SRR8953241–SRR8953294.

## Results

### BB Symptoms on Sampled Rice Leaves

To evaluate the generalized effect of BB on the endophytic microbiota of rice leaves, we selected nine varieties of rice grown in different paddy fields (**Table S1**). The tarnish-gray to white lesions along leaf veins produced by BB were observable on the diseased rice leaves (**Supplementary Figure S1A**). The leaves of BB-susceptible rice varieties (A and B) developed longer lesions (>20 cm in length) than those of the BB-resistant varieties (C and D) (p ≤ 0.001) and the Chinese hybrid rice varieties (E, F, G, H, and I) (nonsignificant, p > 0.05) did (**Supplementary Figure S1A**). The bacterial strains isolated from BB-diseased leaves formed yellow colonies on the PSA plates (**Supplementary Figure S1B**), which were identified as *Xoo* based on BLASTn search results of the full length 16S rRNA gene (**Supplementary Figure S1C**), confirming that the proper causal agent of BB, *Xoo*, caused the infection on sampled BB leaves in this study.

### Metabarcoding Sequencing Data

A total of 4,223,941 high quality bacterial 16S rRNA gene V4–V5 markers were clustered into 855 OTUs (MEAN ± SD = 90 ± 142 per sample) at 97% sequence identity cut-off. Each sample had 78,221 ± 5,691 reads. Approximately 74.7% of the sequences were assigned to chloroplasts (2,776,979 reads in 18 OTUs) or mitochondria (378,037 reads in 6 OTUs), which were removed. The remaining 831 OTUs were subjected to the characterization of the endophytic bacterial communities. For the fungal communities, 3,814,684 ITS1 reads passed quality control (70,642 ± 14,949 reads per sample), which were grouped into 1,408 OTUs (226 ± 114 per sample) at 97% similarity. Both OTU tables were rarefied (sample size n = 61,415 reads for bacteria and n = 25,549 reads for fungi) prior to subsequent analyses.

### Endophytic Microbiome Diversity Influenced by Rice Variety, BB-Resistance Level and BB Occurrence

We first determined if the endophytic microbiome of rice leaves was variety-specific as previously described (Walitang et al., 2018) and if it was impacted by BB occurrence. MLR analysis (**Supplementary Table S1**) showed that rice variety had significant impact on the alpha-diversity indices, including Shannon and Simpson-based true diversities (Jost, 2006) and Chao1, of both the bacterial and fungal communities (ANOVA p < 0.05). However, at a significance level of alpha = 0.05, pairwise comparison based on Tukey’s Honest test showed little differences in these diversity indices between majority of the rice genotypes (**Supplementary Table S1**). Interestingly, the Shannon-based true diversity of fungal communities was significantly lower in BB-diseased leaves (TukeyHSD adjusted p < 0.05) (**Supplementary Figure S2A**), while the alpha-diversity of the bacterial communities based on Chao1 index was significantly higher in BB-diseased leaves (adjusted p < 0.001) (**Supplementary Figure S2B**), irrespective of the BB-resistance level. This may suggest that BB triggered the requisition of specific bacterial taxa during disease development.

ANOSIM analysis suggested that there was a significant shift in the compositional structure of the bacterial (ANOSIM p = 0.001, R = 0.097) and the fungal (p = 0.001, R = 0.271) communities between the healthy and the BB-diseased leaves. Permutation test for homogeneity of group dispersions showed that the fungal communities of BB-susceptible varieties were more variable (p ≤ 0.01) than those of the resistant varieties. By contrast, group dispersions of the bacterial communities were homogeneous (p = 0.14). The RDA models built by stepwise regression showed that the 52.9% variance in bacterial community composition was explained collectively by rice variety (25.7%), BB occurrence (4.5%), and their joint effect (22.7%). The same set of factors explained 43.7% variance in fungal community composition, with rice variety being the most important factor. These results suggest that rice plant genotype indeed played important roles in shaping the endophytic bacterial and fungal communities. By including several different rice varieties in this study, we were able to identify microbial indicators associated specifically with BB occurrence irrespective of the rice genotype or BB-resistance level.

### Endophytic Bacterial Community Composition

**Figure 1:**
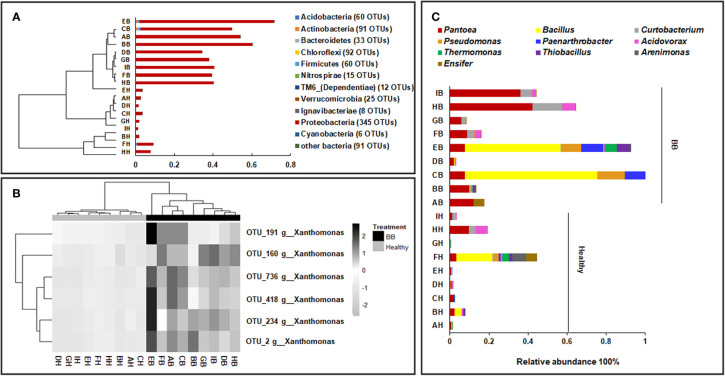
Compositional structure of the endophytic bacterial communities of rice leaves. **(A)** The hierarchical clustering of samples based on the relative abundance of dominant bacterial phyla; **(B)** The abundance of the *Xanthomonas* OTUs in BB-diseased and healthy leaves; **(C)** The relative abundance of the dominant bacterial genera recovered from BB-diseased or healthy leaves. AH-IH, healthy leaves of rice cultivars; AB-IB, BB leaves of rice cultivars used in this study.

Among the 831 bacterial OTUs, 345 (41.5%) were assigned to Proteobacteria (Healthy: 3.5 ± 2.7%; BB: 47 ± 11.8%, *p* ≤ 0.05) (**Figure 1A**), including six belonging to *Xanthomonas* (Healthy: 3.3 ± 2.5%; BB: 46.3 ± 11.4%) (**Figure 1B**). Actinobacteria (Healthy: 0.03 ± 0.03%; BB: 0.23 ± 0.23%) and Acidobacteria (Healthy: 0.01 ± 0.03%; BB: 0.08 ± 0.08%) were also significantly more abundant in BB than in healthy leaves (**Figure 1A**). Only 313 OTUs were assigned to known bacterial genera. In particular, the representative sequence of OTU_2 (45.8% ± 11.5% in BB leaves), had 100% identity with several *Xoo* strains, including *Xoo* PXO99^A^, *Xoo* PXO061 (CP033187.1), *Xoo* PXO513 (CP033188.1), based on the result of a BLASTn search against the GenBank nt database (**Figure 1B**; **Supplementary Table S2**). The other five *Xanthomonas* OTUs were low in abundance (<0.3%) and had 96–98% identity with *Xoo* strain (**Figure 1B** and **Supplementary Table S2**). All six *Xanthomonas* OTUs were also detected with low abundance (<3.2%) in healthy rice leaves. Other abundant genera (>1%) recovered included *Bacillus*, *Pantoea*, *Curtobacterium*, *Arenimonas*, *Paenarthrobacter*, *Pseudomonas*, *Paenibacillus*, *Acidovorax*, *Thermomonas*, and *Gemmobacter* (**Figure 1C**). Among these genera, *Pantoea*, *Curtobacterium*, and *Pseudomonas* were significantly more abundant in BB leaves than in healthy leaves (*p* ≤ 0.05). We further identified 83 bacterial OTUs enriched significantly in BB than in healthy leaves (*p* < 0.05) (**Supplementary Table S3** and **Figure S3A**). Interestingly, there no bacterial OTU was significantly enriched in healthy leaves than in BB leaves (**Supplementary Table S3**). These results further confirmed that BB induced an increase in the diversity of endophytic bacterial community, as also demonstrated by the MLR models (**Supplementary Figure S1**).

By comparing with the FAPROTAX database, 249 OTUs were classified into 57 functional groups (**Supplementary Table S4**). The members of Proteobacteria and Actinobacteria were involved with diverse ecological functions (**Supplementary Figure S4A**). A total of 39 bacterial functional groups were significantly more abundant in BB than in healthy leaves (*p* < 0.05), such as those affiliated with chemoheterotrophy (including aerobic chemoheterotrophy), fermentation, and nitrate reduction, or being identified as plant pathogens. By contrast, functional groups involved with hydrocarbon degradation, ligninolysis, aromatic hydrocarbon degradation, and aliphatic nonmethane hydrocarbon degradation were more abundant in healthy leaves than in BB leaves (**Supplementary Figure S4B**; **Supplementary Table S4**).

### Endophytic Fungal Community Composition

**Figure 2:**
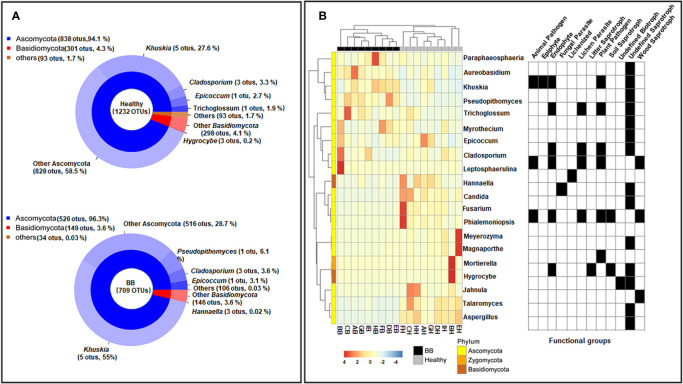
Compositional structure of the endophytic fungal communities of rice leaves. **(A)** The relative abundance of fungal phyla (top) and genera (bottom). **(B)** Hierarchical clustering and annotation of the 20 most abundant fungal genera in healthy or symptomatic leaves, based on their relative abundance in healthy and BB-diseased rice leaves. The annotation panel on the right indicated the functional guilds each genus being assigned to by FUNGuild.

The 1,408 fungal ITS1 OTUs (healthy: 1,232; BB: 709) were assigned to six phyla, 30 classes, 89 orders, 174 families, and 253 genera. Not surprisingly, Ascomycota (healthy: 94.1 ± 7.1%; BB: 96.3 ± 8%) and Basidiomycota (healthy: 4.3 ± 3.8%; BB: 3.6 ± 8%) were most abundant in all samples; however, Ascomycota was significantly more abundant in BB leaves while Basidiomycota was significantly more abundant in healthy leaves (*p* < 0.05) (**Figure 2A**). Among the 20 most abundant genera (**Figure 2B**), *Khuskia*, *Pseudopithomyces*, *Cladosporium*, *Leptosphaerulina*, *Trichoglossum*, *Aureobasidium*, *Myrothecium*, and *Paraphaeosphaeria* were significantly more abundant in BB than in healthy leaves, while *Fusarium*, *Meyerozyma*, *Magnaporthe*, *Phialemoniopsis*, *Talaromyces*, *Aspergillus*, *Jahnula*, and *Candida* showed the opposite trend (*p* < 0.05). We further identified 376 fungal OTUs that differed significantly in abundance between the BB and healthy leaves (*p* < 0.05) (**Supplementary Table S5**), among which, the OTUs with the relative abundance > 0.1% are shown in **Supplementary Figure S3B**.

FUNGuild categorized 750 ITS1 OTUs into four trophic modes (**Supplementary Figure S5A**) and 27 ecological guilds (**Supplementary Table S6**). The saprotrophs were found to be significantly more abundant in BB than in healthy leaves (healthy: 43.1% ± 22.6%, BB: 72.2% ± 19.6%; p < 0.05) (**Supplementary Figure S5B**). Fungi categorized as endophytes, plant pathogens, and/or lichen parasites were also abundant (>10%), however, did not differ significantly in abundance between BB and healthy leaves (**Supplementary Figure S5B**).

### Bacterial Endophytes From *Xoo*-Infected Rice Leaves

Although the functions of many endophytes in healthy plants have been studied in detail, we still know little about the roles of the endophytes in the occurrence of plant disease. The community analysis has showed that bacterial taxa are enriched in BB rice leaves. To further investigate the potential function of BB-induced accumulation of bacterial endophytes, we isolated bacterial strains from BB-diseased leaves of rice grown in geographically distant farmer’s fields in Yunnan and Guangdong provinces. In total, we isolated 30 bacterial strains from surface-sterilized BB leaf tissues (details in **Table 2**). Through BLASTn search of the full-length 16S rRNA gene sequences against the GenBank nt database, 14 isolates belong to the Actinobacteria, 12 to Proteobacteria, and four to the Firmicutes phylum, including the strains of the bacterial genera (*Pantoea*, *Pseudomonas*, and *Curtobacterium*) that were significantly enriched in BB-diseased leaves (**Table 2**).

**Figure 3:**
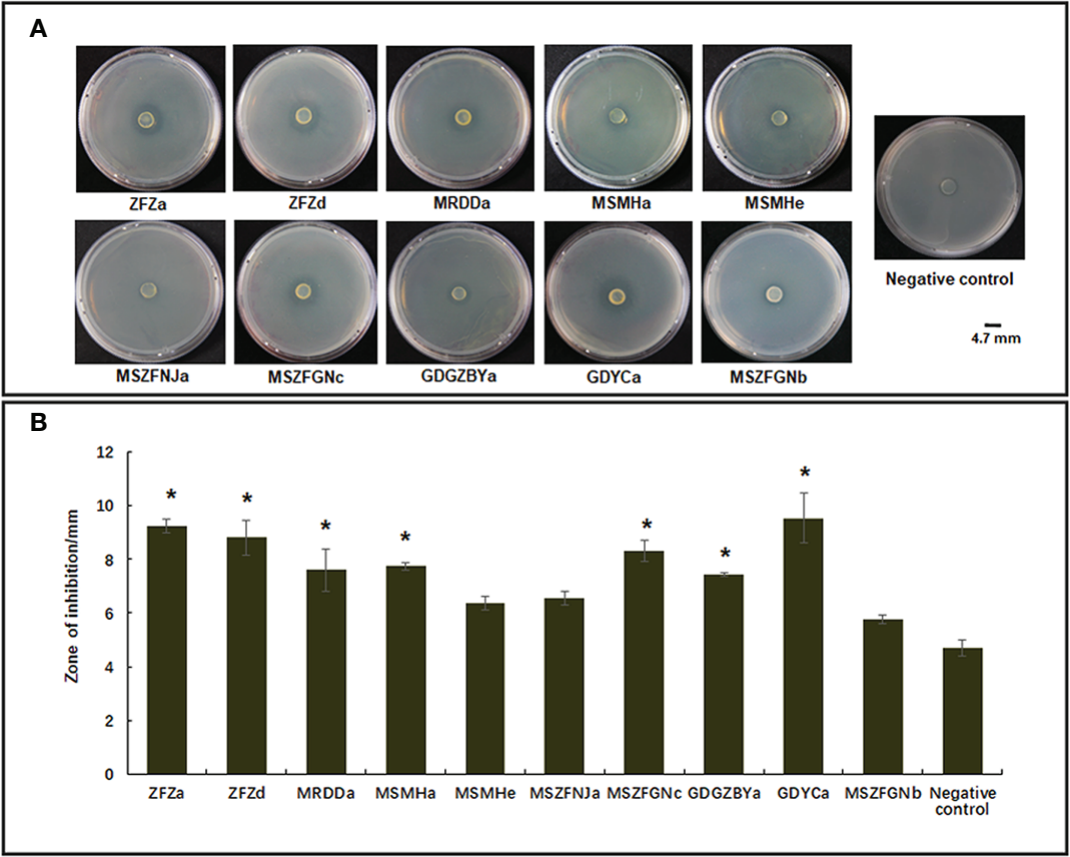
Anti-*Xoo* activities of the endophytic strains isolated from BB-diseased rice leaves. **(A)** Representative results of zone of inhibition tests. **(B)** Diameter of zone of inhibition. The error bar represents standard deviation for three independent replicates. NC, negative control. * indicates p < 0.05 by t-test.

**Figure 4:**
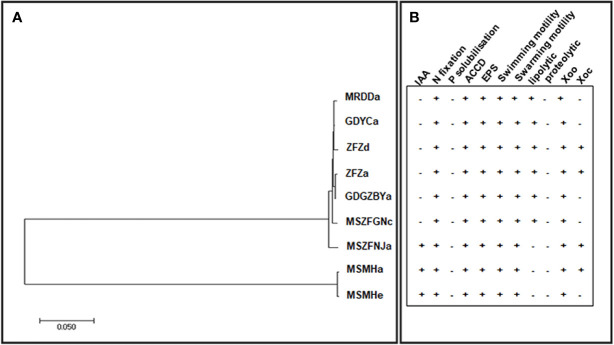
The NJ tree based on full-length 16S rRNA gene and the PGP traits of the endophytic *Pantoea* sp. isolates. **(A)** The NJ tree reconstructed based on the full-length of 16S rRNA gene sequences of *Pantoea* sp. strains by MEGA (version 7.0.21). **(B)** Characterization of *in vitro* plant growth-promoting traits. IAA, indole acetic acid production; N, nitrogen fixation; P, phosphorous solubilization; ACCD, ACC Deaminase activity; EPS, exopolysaccharide production. Swimming and swarming motility, lipolytic and proteolytic activity, and antibacterial activity against *Xoo* and *Xoc*. Each experiment was performed with three biological replicates. Positive detection (+), negative detection (−).

Previous studies have identified diverse bacterial endophytes with PGP traits as potential antagonists to plant pathogens (Compant et al., 2005; Vannier et al., 2019). Therefore, we first screened bacterial endophytic isolates for antibacterial activity against *Xoo*. The zone of inhibition tests showed that nine *Pantoea* strains (ZFZa, ZFZd, MRDDa, MSMHa, MSMHe, MSZFNJa, MSZFGNc, GDGZBYa, and GDYCa) and one *Pseudomonas* strain (MSZFGNb) inhibited the growth of *Xoo* in the PSA plates at different degrees (**Figures 3A, B**), while the other endophytic bacterial strains did not show any inhibitory effect against *Xoo* (data not shown). The NJ tree of the full-length 16S rRNA gene sequences clustered seven *Pantoea* sp. strains together (group I), while the other two strains formed group II (**Table 2** and **Figure 4A**). All nine *Pantoea* sp. strains exhibited activities in nitrogen fixation, ACC deaminase activity, EPS production, and cell motility, but not in phosphate solubilization and secretion of proteolytic enzyme (**Figure 4B** and **Supplementary Figure S6**). In particular, three strains (MSZFNJa, MSMHa, and MSMHe) produced IAA, while another six strains secreted lipolytic enzymes. In addition, four *Pantoea* sp. strains (ZFZa, ZFZd, MSZFNJa, and MSMHa) showed inhibitory effects on *Xanthomonas oryzae* pv. *oryzicola* (*Xoc*), which is the causal agent of bacterial leaf streak of rice (**Figure 4B** and **Supplementary Figure S6**).

### Endophytic *Pantoea* sp. Strains Suppressed BB Disease in Rice

**Figure 5:**
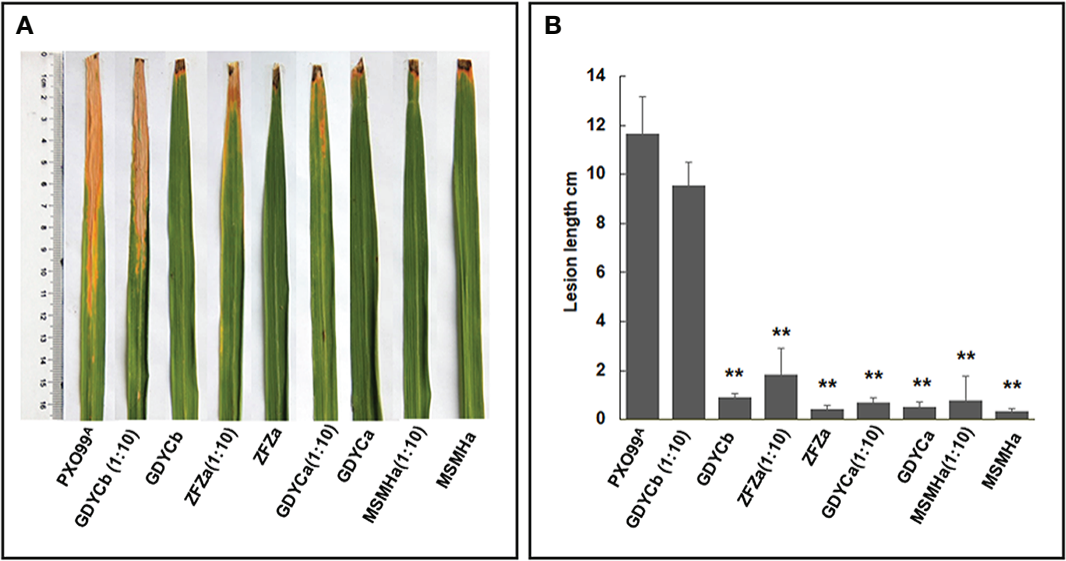
*In-planta* BB suppression assays. **(A)** Each leaf was inoculated with *Xoo* PXO99^A^, *Pantoea* sp. isolates, *Curtobacterium* sp. isolate, the mixture of *Pantoea* sp. isolate or *Curtobacterium* sp. Isolate, and *Xoo* PXO99^A^ at 1:10 ratio using the leaf clipping method. BB symptoms were observed on the 14th day after inoculation. **(B)** The length of the disease lesion on each leaf. The error bar represents standard deviation of the lesion lengths recorded from ≥10 leaves. ** indicates p < 0.01 by t-test.

Among the nine bacterial strains that inhibited the growth of BB causal agent *Xoo* on plates, we selected two *Pantoea* strains (ZFZa and GDYCa) from group I and one (MSMHa) from group II (**Table 2** and **Figure 4A**) to determine their suppressive effects on BB of rice by co-inoculation of each strain with *Xoo*. We also included an endophytic *Curtobacterium* sp. GDYCb which did not inhibit the *in vitro* growth of *Xoo* as a negative control. No visible phenotypic changes or lesions were observed when the rice leaves were inoculated with endophytic bacterial strain only (**Figures 5A, B**). When *Xoo* (strain PXO99^A^) was co-inoculated with each *Pantoea* sp. strain, the lengths of the BB lesions were significantly shorter than those of the leaves that were inoculated with *Xoo* PXO99^A^ only (p ≤ 0.01). *Curtobacterium* sp. GDYCb (negative control) did not inhibit BB development caused by *Xoo* PXO99^A^ (**Figure 5B**). These results suggested that the endophytic *Pantoea* sp. isolates were nonpathogenic to rice and suppressed BB-disease development in rice.

## Discussion

Microbial endophytes form symbiotic associations with plants and often promote the performance, growth, and/or health of the host by suppressing disease, enhancing nutrient uptake and use, or improving resistance to abiotic and biotic stresses (Lugtenberg et al., 2016; Strobel, 2018). The effects of disease on the phytomicrobiome have been studied in recent years (Yang et al., 2016; Wang et al., 2017; Zhang et al., 2017; Shi et al., 2019). However, few studies have investigated the effects of BB disease on the endophytic bacterial and fungal communities of rice. In this study, we evaluated the impact of rice genotype and BB-resistance level on the endophytic bacterial and fungal communities of rice leaves, revealed BB-induced shift in community diversity and compositional structure, and investigated the potential roles of endophytic bacterial isolates for the control of BB in rice.

It has been reported that some plant diseases have led to a reduction in host endophytic microbial community diversity, including Huanglongbing (Citrus greening) disease on citrus and Clubroot caused by *Plasmodiophora brassicae* on cruciferous (Breidenbach et al., 2015; Venkatachalam et al., 2016)). We observed a BB-associated increase in the diversity of the endophytic bacterial communities through metabarcoding approach and community analysis (**Supplementary Figure S2**). The increase of the diversity of the endophytic bacteria was found in pinewoods with pine wilt disease (Proenca et al., 2017). The authors postulated that the causal nematode, *Bursaphelenchus xylophilus*, might have repressed the host defense system and therefore allowed excessive internal colonization of soil microorganisms. It suggested that the enrichment of some endophytes might be beneficial for the host and acquired by the host in response to the infection of the pathogen.

Here, we revealed that *Pantoea*, *Curtobacterium*, and *Pseudomonas* are ubiquitous and significantly enriched in BB-diseased leaves, irrespective of the rice genotype or BB-resistance level. *Pantoea* spp., *Curtobacterium* spp., and *Pseudomonas* spp. strains have been found inside healthy rice plants and have shown beneficial activities for plant health and development (Mano and Morisaki, 2008; Morontabarrios et al., 2018). However, the roles of these endophytes in the occurrence of BB remain unclear. *Pantoea* sp. isolates from BB leaves had antagonistic activity towards *Xoo in vitro* and significantly suppressed the lesion development in the occurrence of BB. In addition, these *Pantoea* sp. strains were not able to initiate BB of rice, although strains of *P. agglomerans* and *P. ananatis* isolated from rice had previously been identified as pathogens causing grain discoloration or leaf blight of rice (Lee et al., 2010; Mondal et al., 2011). Therefore, we speculate that *Pantoea* sp. strains function as antagonist of pathogen rather than the partner of pathogen leading to serious disease in BB occurrence. *Pantoea* sp. strains were isolated from BB-diseased leaves of different rice varieties grown in different paddy fields, suggested that their function in BB occurrence might be universal, and such enrichment might be induced by BB development. However, *Pantoea* sp. strains could not suppress the occurrence of BB in the field possibly because of the lower concentration of these strains compared with the pathogen in heavily infested BB leaves. Further identification of these *Pantoea* spp. at higher taxonomic resolution by sequencing their genome and increasing their colonization in rice will promote *Pantoea* spp. as a biocontrol measure for BB disease. The antagonism of *Pseudomonas* sp. strains against BB of rice have been evaluated *in vitro* and in field tests, while endophytic *Curtobacterium* sp. have been assessed for their potential as biopesticides for disease control and plant growth promotion (Velusamy et al., 2006; Yasmin et al., 2017). Although *Curtobacterium* spp. and *Pseudomonas* spp. strains from BB leaves did not show antagonistic activity towards *Xoo*, they might perform unknown functions during BB occurrence.

Besides endophytic bacterial species, fungal endophytes also play a key role in promoting plant performance and resistance/tolerance to biotic and abiotic stresses (Lugtenberg et al., 2016; Tetard-Jones and Edwards, 2016). Previous studies have revealed a decrease in the overall diversity of endophytic fungal community but an enrichment of potentially beneficial antagonists in Fusarium-Head-Blight-diseased wheat spikes (Rojas et al., 2019). The endophytic fungal communities in *Xoo*-infected leaves were not only lower in diversity, but also, perhaps more interestingly, enriched with some potential pathogenic or mycotoxin-producing fungi, *e.g. Fusarium* sp. (Desjardins et al., 2000; Lei et al., 2019), *Magnaporthe* sp. (Pennisi, 2010), and *Aspergillus* sp. (Reddy et al., 2009). The latter may suggest more competition between these fungal pathogens and *Xoo* in healthy or asymptomatic leaves. However, nonpathogenic strains of *Fusarium*, *Aspergillus*, and *Phialemoniopsis* have been assessed for antagonistic activities against plant pathogens including *F. oxysporum*, *Colletotricum gloeosporioides*, and *Sclerotium rolfsii* (Tayung and Jha, 2010; Waqas et al., 2015; Mastan et al., 2019). Therefore, it may be worthwhile to further isolate and determine the identity and virulence of these fungal strains. By contrast, Ascomycota spp. in *Aureobasidium, Epicoccum*, *Khuskia*, *Leptosphaerulin*, *Myrothecium*, *Paraphaeosphaeria*, *Pseudopithomyces*, and *Trichoglossum* were recovered more in BB symptomatic leaves relative to asymptomatic or healthy leaves (**Figure 2**). *Khuskia* spp. and *Leptosphaerulina* spp. are capable of producing lingnases, xylanases, and/or Mn-peroxidase (Slavikova et al., 2002; Wu et al., 2013; Sajben-Nagy et al., 2014). Therefore, the infection of *Xoo* may create a microenvironment that is suitable for some saprophytic Ascomycota spp. governing decomposition of plant residue. It is noteworthy that some widely distributed endophytic fungi, *e.g. Epicoccum* spp. (*e.g. E. nigrum*), *Aureobasidium* spp. (*e.g. A. pullulans*), and *Phaeosphaeria* spp., have shown antagonistic activities against plant pathogens (*e.g. Botrytis cinerea*, *Rhizoctonia solani, Plasmopara viticola*, and *Fusarium graminearum etc*.) by competing for nutrients or producing a wide array of secondary metabolites including antimicrobials (Martini et al., 2009; Herrera et al., 2010; Lahlali and Hijri, 2010; Brum et al., 2012; Varanda et al., 2016).

*Xoo* is a pathovar of *Xanthomonas oryzae* and is the causative agent of BB on rice (Nino-Liu et al., 2006). Our study identified six *Xanthomonas* OTUs (**Supplementary Table S2**), among which, only one (OTU_2) was most abundant in BB-diseased leaves and was classified as the BB causal agent based on 16S rRNA gene sequence that shared 100% similarity with those of *Xoo* type strains. The fact that OTU_2 was also recovered from asymptomatic rice leaves at much lower abundance suggests an association between the abundance of causal agents and the symptom severity of plant diseases, as also has been addressed by other studies (Manching et al., 2014; Blaustein et al., 2017; Shi et al., 2019). All other five *Xanthomonas* OTUs recovered in this study were in very low abundance (<0.3%) in BB leaves and shared high (96–98%) identity with *Xoo* strains (**Supplementary Table S2**). Many studies have shown that pathogens often do not act alone during disease progression; the occurrence, development, and severity of which are results of synergistic interactions among multiple phytopathogens (Lamichhane and Venturi, 2015). Moreover, the mechanisms for competitive or complaisant interactions among microbes in plant disease development are still under-studied, but evidence has suggested that the host defenses and immunity may be activated or suppressed by avirulent, beneficial, or pathogenic microbial cohabitants (Lamichhane and Venturi, 2015). The reasons for the existence of multiple *Xoo* strains in BB occurrence are not clear, although some endophytic *Xoo* strains that have been identified from rice seeds were nonpathogenic (Walitang et al., 2018). If *Xoo*-related isolates were nonpathogenic, they may act as “antigens” to induce plant resistance responses and/or compete with pathogens, in this case *Xoo*, for nutrients and/or space, as shown in a study on Verticillium Wilt (Deketelaere et al., 2017).

We acknowledge that a large amount of metabarcodes obtained in this study were classified to plant chloroplast and mitochondria. This is because the 16S rRNA gene region was amplified from the total genomic DNA of rice leaves using the universal primer 515F/907R without blocking primers (Arenz et al., 2015; Morontabarrios et al., 2018) to reduce the co-amplification of plant mitochondrial and chloroplast rDNA (**Figure 2A**). Considering the importance of chloroplasts and mitochondria in host immune defence during pathogen infection (Caplan et al., 2015), the fact that we recovered significantly more copies of plant-derived sequences in healthy leaves than in BB leaves, perhaps reflects a decrease or dysfunction of these organelles in discolored lesions where the genomic DNA was extracted from symptomatic leaf samples.

## Conclusion

Plants benefit from symbiotic relationships with an endophytic microbiome, but how individual endophytes or the endophytic microbial community as a whole respond to disease development and confer pathogen resistance to the host plant is still under-studied. The current study showed an array of adverse effects of *Xoo* infection on the endophytic microbiota of rice leaves, such as a decrease in the fungal community diversity. However, *Xoo* invasion may also activate the immune response of the plant by acquiring beneficial microbes, such as PGP bacteria and nonpathogenic close relatives to the causal agents, which in turn promote the health and disease suppression of the host. Therefore, deciphering the changes in compositional structure, function, and multilateral interactions of the endophytes under pathogen attack paves the way for identifying naturally occurring biocontrol agents for disease management and control.

## Data Availability Statement

The datasets generated for this study can be found in the raw Illumina HiSeq paired-end sequences which have been deposited at the NCBI’s sequence read archive (SRA) in BioProject ID PRJNA534010 with accession numbers SRR8948950–SRR8949003 and PRJNA533998 with accession numbers SRR8953241–SRR8953294.

## Author Contributions

FY and WC designed the experiments. JZ performed the experiments. JZ, FY, HZ, GJ, LZ, YL, and WC analyzed the data. FY, WC, and WF wrote the manuscript. All authors contributed to the article and approved the submitted version.

## Funding

This research was funded by the National Key R&D Program of China (2016YFD0300701). The Agricultural Science and Technology Innovation Program (ASTIP) from Chinese Academy of Agricultural Sciences and The Agriculture and Agri-Food Canada’s (AAFC) Foreign Research Participant Program supported FY’s research at the Ottawa Research and Development Centre, Ottawa, ON, Canada. The computational infrastructure was partially supported by AAFC-funded A-base projects J-001012, J-000985, and J-001272, as well as the Government of Canada’s Genomics Research and Development Initiative (GRDI) Shared Priority Project—EcoBiomics (J-001263).

## Conflict of Interest

The authors declare that the research was conducted in the absence of any commercial or financial relationships that could be construed as a potential conflict of interest.
