# Supplementary material for: Bacterial Blight Induced Shifts in Endophytic Microbiome of Rice Leaves and the Enrichment of Specific Bacterial Strains With Pathogen Antagonism
Source: Front Plant Sci. 2020 Jul 23;11:963. doi: 10.3389/fpls.2020.00963 (PMC7390967; doi:10.3389/fpls.2020.00963)
Supplement: Supplementary file 1 [file Image_1.pdf]

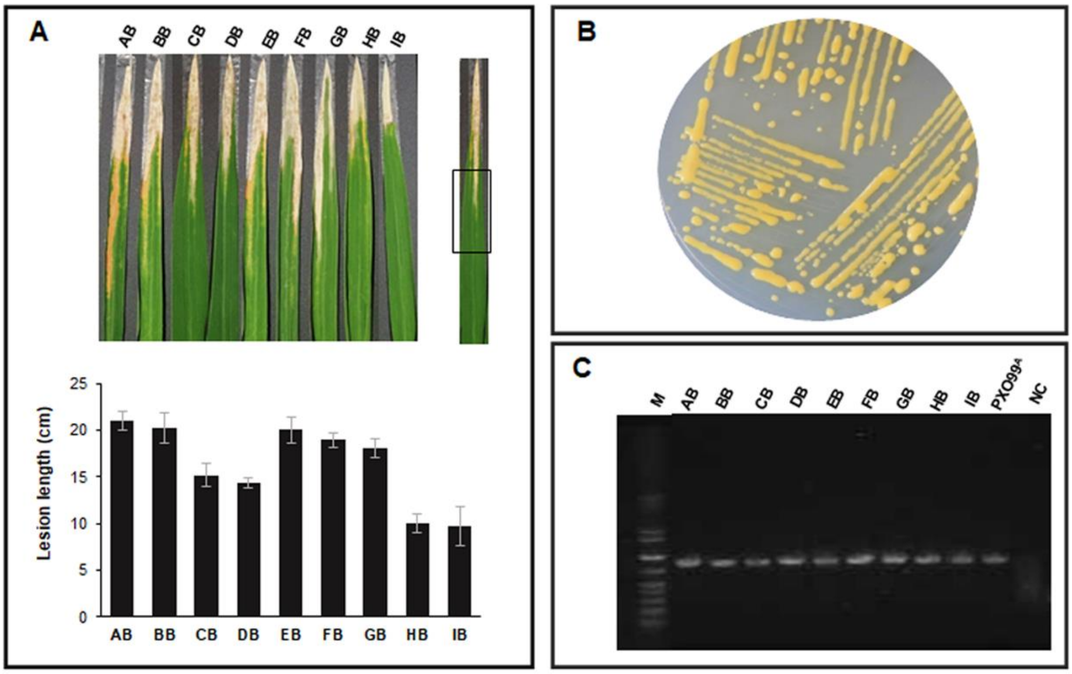

Supplementary Figure S1. Symptomatic leaves of bacterial-blight (BB)-diseased rice plants and PCR detection of *Xoo*. (A) Symptoms of BB-diseased leaves collected from the fields; The diseased-healthy junction of rice leaves in the white frame represent where the DNA was extracted for profiling the endophytic microbiota. (B) The colony of *Xanthomonas oryzae* pv *oryzae* (*Xoo*) isolated from BB leaves. (C) PCR amplification of *Xoo* by the primers OSF1/OSR1. From left to right, Lane M: DNA ladder markers; Lanes 2–9: leaf samples; Lane 10: *Xoo* strain PXO99^{A}. AB-IB means the BB leaves of different rice cultivars used in this study.
