# Supplementary material for: Bacterial Blight Induced Shifts in Endophytic Microbiome of Rice Leaves and the Enrichment of Specific Bacterial Strains With Pathogen Antagonism
Source: Front Plant Sci. 2020 Jul 23;11:963. doi: 10.3389/fpls.2020.00963 (PMC7390967; doi:10.3389/fpls.2020.00963)
Supplement: Supplementary file 2 [file Image_2.pdf]

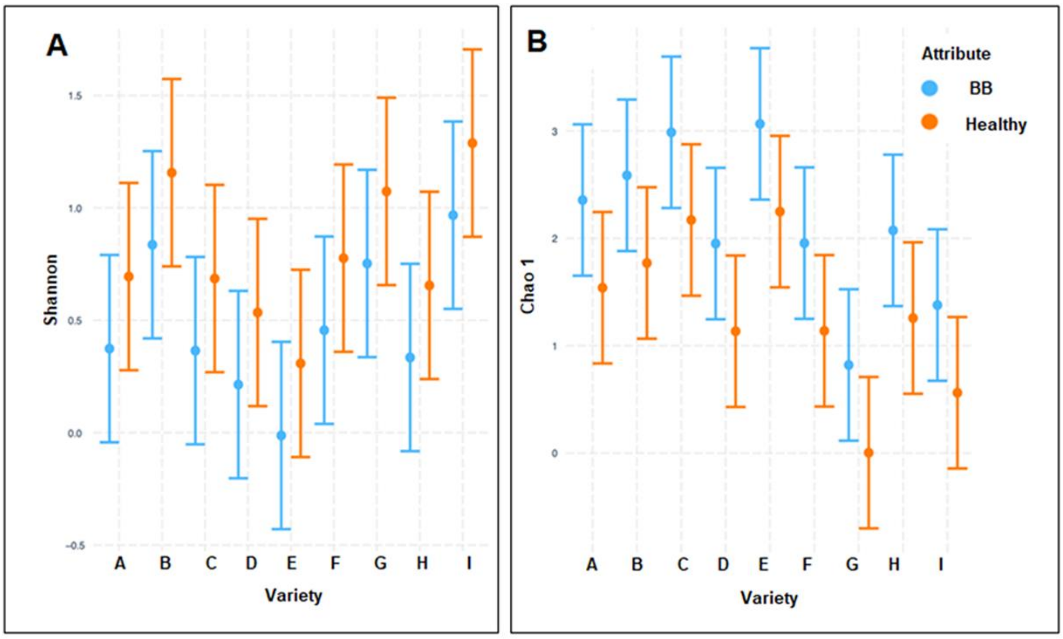

Supplementary Figure S1. The alpha-diversity indices of the endophytic microbial communities. A) the Shannon-based true diversity of fungal communities was significantly lower in BB-diseased leaves (TukeyHSD adjusted $p < 0.05$). B) The Chao1 index of the bacterial communities was significantly higher in BB-diseased leaves (adjusted $p < 0.001$).
