# Supplementary material for: Bacterial Blight Induced Shifts in Endophytic Microbiome of Rice Leaves and the Enrichment of Specific Bacterial Strains With Pathogen Antagonism
Source: Front Plant Sci. 2020 Jul 23;11:963. doi: 10.3389/fpls.2020.00963 (PMC7390967; doi:10.3389/fpls.2020.00963)
Supplement: Supplementary file 3 [file Image_3.pdf]

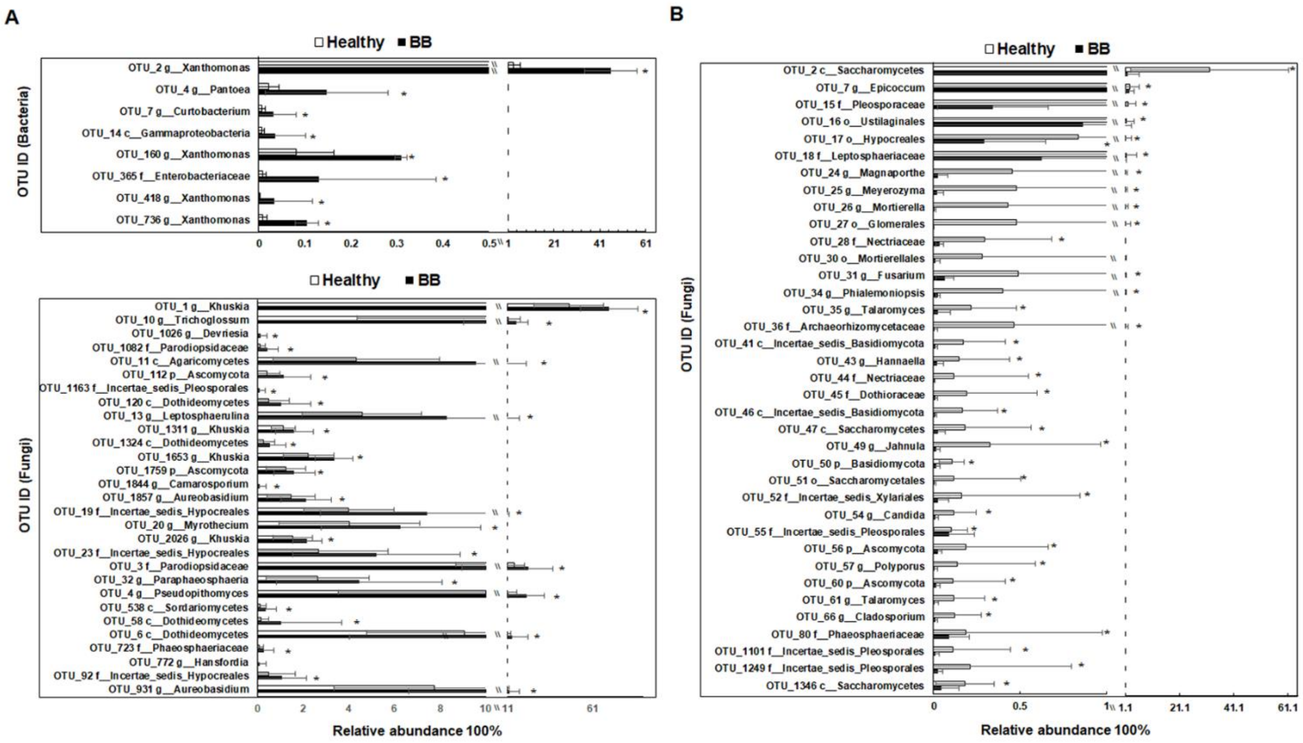

Supplementary Figure S3. The relative abundance of OTUs in BB-diseased and healthy or asymptomatic leaves. (A) Bacterial (top) and fungal (bottom) OTUs significantly enriched in BB relative to healthy leaves. (B) OTUs significantly depleted in BB relative to asymptomatic/healthy leaves. (wilcox.test, *p* < 0.05)
