# Supplementary figures and images for: Bacterial Blight Induced Shifts in Endophytic Microbiome of Rice Leaves and the Enrichment of Specific Bacterial Strains With Pathogen Antagonism
Source: Front Plant Sci. 2020 Jul 23;11:963. doi: 10.3389/fpls.2020.00963 (PMC7390967; doi:10.3389/fpls.2020.00963)

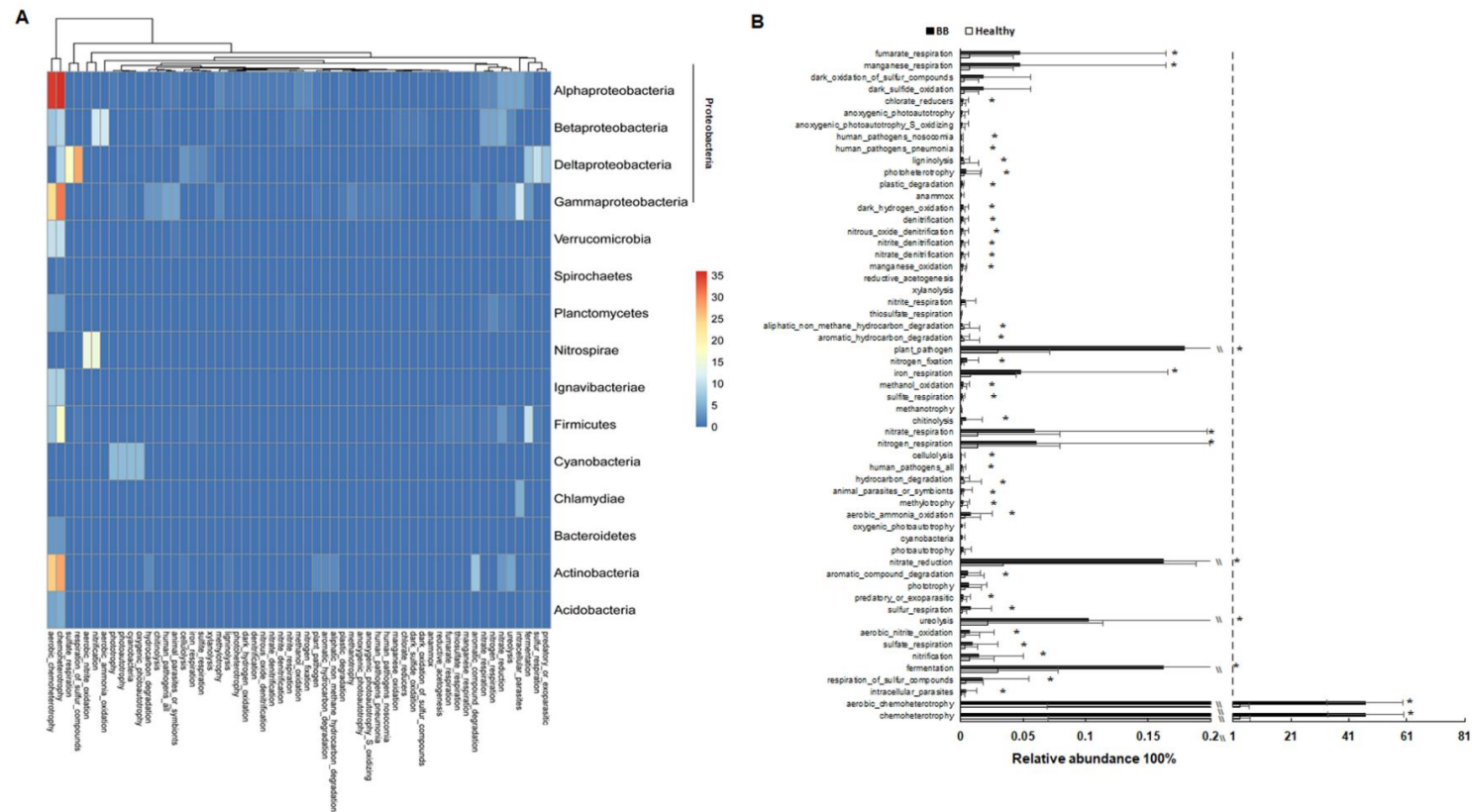

Supplement: Supplementary file 4 [file Image_4.pdf]
