# Supplementary material for: Bacterial Blight Induced Shifts in Endophytic Microbiome of Rice Leaves and the Enrichment of Specific Bacterial Strains With Pathogen Antagonism
Source: Front Plant Sci. 2020 Jul 23;11:963. doi: 10.3389/fpls.2020.00963 (PMC7390967; doi:10.3389/fpls.2020.00963)
Supplement: Supplementary file 5 [file Image_5.pdf]

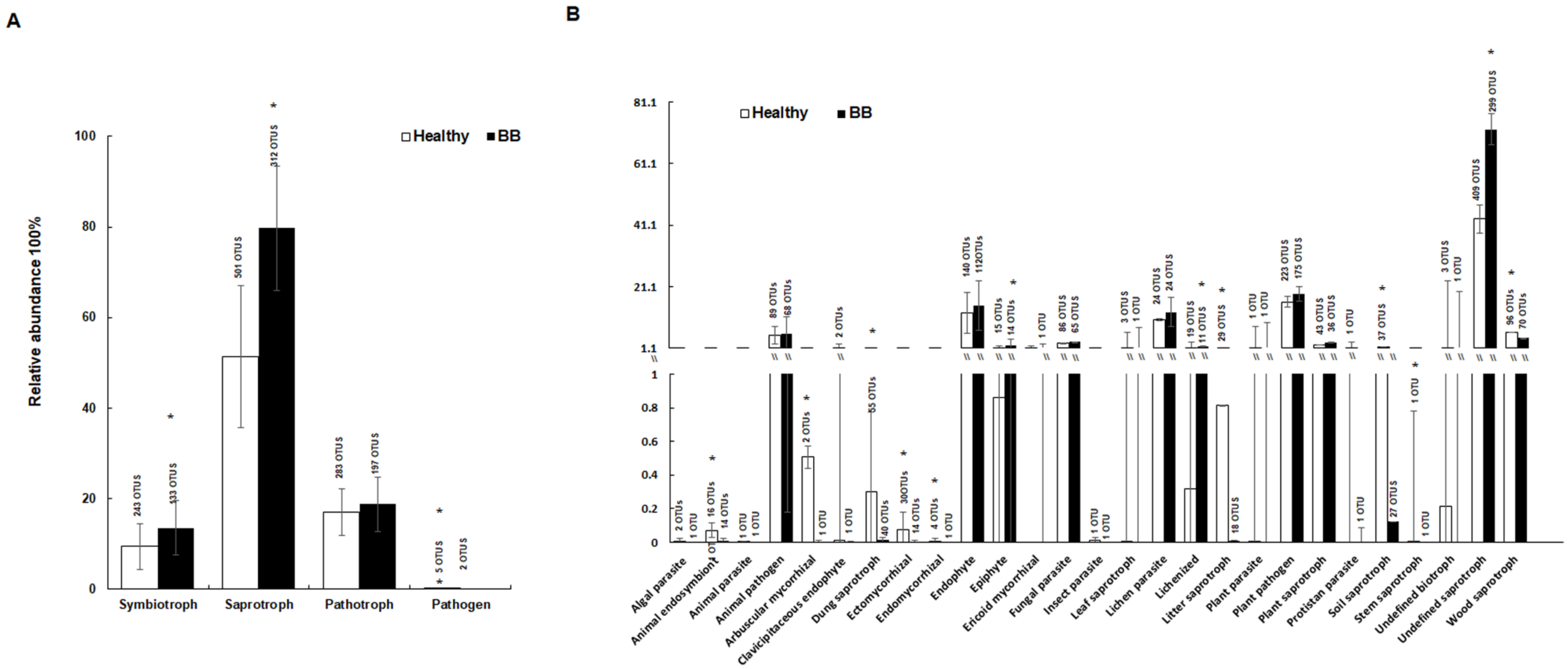

Supplementary Figure S5. Functional annotation of fungal and bacterial OTUs in BB-diseased and asymptomatic/healthy leaves. The relative abundances and numbers of fungal OTUs in (A) four trophic modes or B) fungal functional groups. * Wilcox test, $p < 0.05$.
