# Supplementary material for: Bacterial Blight Induced Shifts in Endophytic Microbiome of Rice Leaves and the Enrichment of Specific Bacterial Strains With Pathogen Antagonism
Source: Front Plant Sci. 2020 Jul 23;11:963. doi: 10.3389/fpls.2020.00963 (PMC7390967; doi:10.3389/fpls.2020.00963)
Supplement: Supplementary file 6 [file Image_6.pdf]

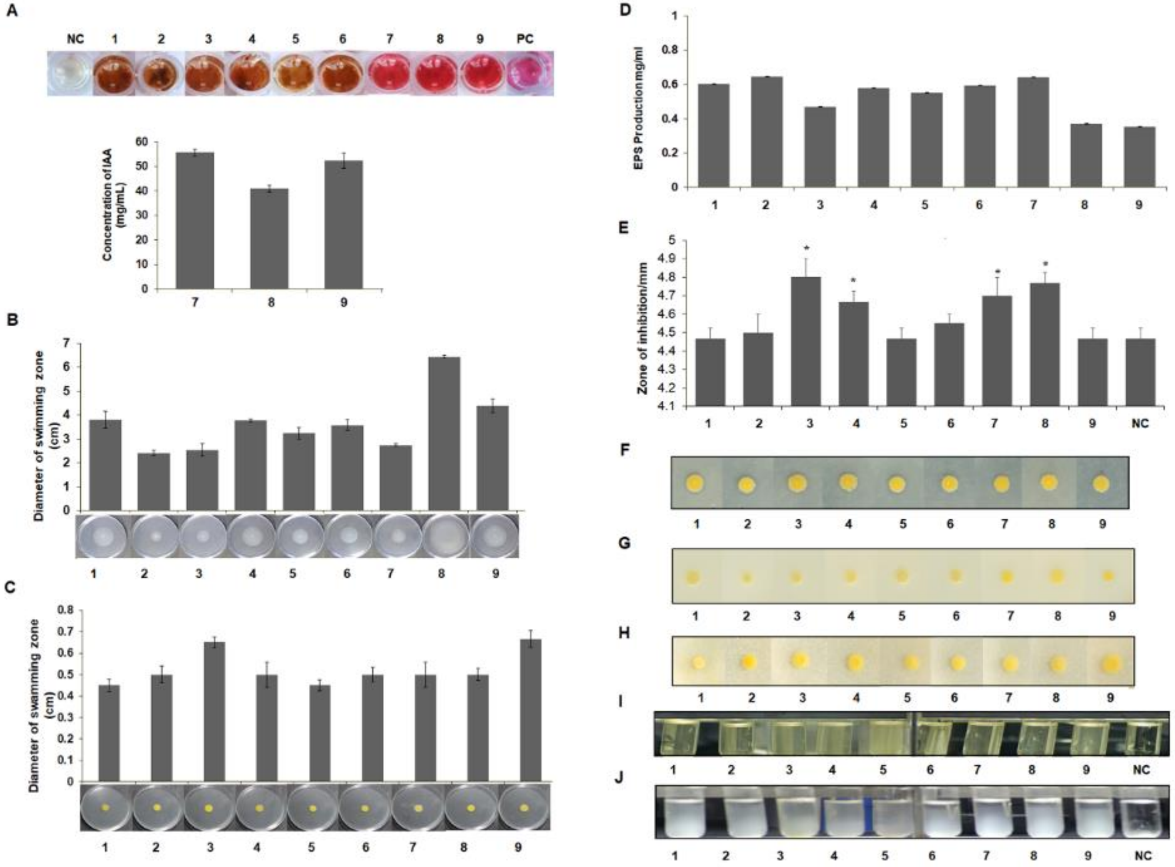

Supplementary Figure S6. Evaluation of plant growth promoting traits of nine *Pantoea* sp. isolates. (A) Three *Pantoea* sp. strain (MSZFNJa, MSMHa and MSMHe) produced IAA. All *Pantoea* sp. isolates displayed (B) swimming on the semi-solid plate containing 0.2% agar, (C) swarming motility on the semi-solid plate containing 0.5% agar. (D) The EPS production were determined in all *Pantoea* sp. strains by

ethanol precipitation method. (E) Four *Pantoea* sp. strains (ZFZa, ZFZd, MSZFNJa, MSMHa) showed inhibitory effects on *Xanthomonas oryzae* pv. *oryzicola*. (F) Six *Pantoea* sp. strains (MRDDa, GDYCa, ZFZd, ZFZa, GDGZBYa, MSZFGNc) secreted lipolytic enzymes on the mTBA plate with 1% glycerin tributyrate. None of the *Pantoea* sp. strains exhibited (G) proteolytic activity on LB plate with 2% skim milk or (H) phosphate solubilization on the mTBA plate. All *Pantoea* sp. strains showed (I) ACC deaminase activity in ADF liquid media which contains the only nitrogen source of ACC deaminase and (J) nitrogen fixation in nitrogen-free liquid media. Each experiment was performed with three biological replicates. NC, Negative control; 1, MRDDa; 2, GDYCa; 3, ZFZd; 4, ZFZa; 5, GDGZBYa; 6, MSZFGNc; 7, MSZFNJa; 8, MSMHa; 9, MSMHe; PC, Positive control.
